# Changes in cortical activation during proprioceptive stimulation and galvanic vestibular stimulation in healthy individuals and individuals with post-stroke balance disorders: A functional near-infrared spectroscopy study

**DOI:** 10.1016/j.nicl.2025.103822

**Published:** 2025-06-08

**Authors:** Lixia Liu, Jingchuan He, Feiyu Nong, Yalin Huang, Shijun Huang, Xiangyu Qin, Chongwu Xiao, Yaobin Long

**Affiliations:** a Department of Rehabilitation Medicine, The Second Affiliated Hospital of Guangxi Medical University, Guangxi Medical University, Nanning 530007, China; b The Second Clinical Medical College of Guangxi Medical University, Guangxi Medical University, Nanning 530007, China; c Department of Pharmacy, Guangxi Medical University Cancer Hospital, Guangxi Medical University, Nanning 530200, China; d Department of Rehabilitation Medicine, The Second Nanning People’s Hospital, Nanning 530031, China

**Keywords:** Proprioceptive stimulation, Vestibular electrical stimulation, Balance disorders, Functional near-infrared spectroscopy, Stroke

## Abstract

**Introduction:**

Although previous studies have shown that galvanic vestibular stimulation (GVS) and proprioceptive stimulation (PS) can improve balance function in patients with post-stroke balance disorders, the exact central mechanisms remain unclear. This study used functional near-infrared spectroscopy (fNIRS) to investigate differences and changes in cortical activation during PS, GVS, and their combination in healthy individuals and patients with post-stroke balance disorders.

**Methods:**

Sixteen patients with post-stroke balance disorders and twenty healthy controls were enrolled as the patient and healthy groups, respectively. Participants stood on the vibrating platform and underwent three tasks: only GVS (Task 1), only PS (Task 2), and GVS and PS (Task 3). Cortical activation of Somatosensory cortex (S1), Motor cortex (MC), Dorsolateral prefrontal cortex (DLPFC), and Broca’s area (Broca) was collected using fNIRS.

**Results:**

Task 1 did not activate any region of interest (ROI) in either group. Task 2 activated right S1, bilateral MC, DLPFC, and Broca in the healthy group. In the patient group, Task 2 activated right S1 and left MC. In contrast, Task 3 elicited activation of all ROIs in both groups. Cortical activation between the two groups during the three tasks did not show any significant differences.

**Conclusion:**

Compared to GVS or PS alone, combining GVS with PS better improved cortical hemodynamic conditions in patients with post-stroke balance disorders. These findings highlight the clinical potential of GVS and PS in the rehabilitation of patients with post-stroke balance disorders, warranting further exploration.

## 1 Introduction

Stroke is one of the most common neurological diseases (Purroy & Montalà, 2021). According to recent epidemiological studies, stroke is currently the second leading cause of death from non-communicable diseases worldwide, with its incidence increasing annually (Wu & Liu, 2024). A report from the American Heart Association indicates that approximately 7 million people experience a stroke in the United States each year (Benjamin et al., 2019), imposing a major social burden. Stroke causes brain damage and results in a range of functional impairments. About 86 % of stroke survivors experience varying degrees of balance disorders (Cho et al., 2015, Tyson et al., 2006). Effective balance maintenance relies on the ability of the central nervous system to integrate information from various sensory organs, thereby generating appropriate muscle responses for maintaining body balance and stability (Peterka, 2002). After a stroke, the central nervous system often fails to accurately analyze and process these sensory inputs, leading to balance dysfunction (Saraiva et al., 2023).

The most important senses for maintaining body balance are proprioception, vestibular sensation, and vision (Steinberg et al., 2023). Proprioception, also known as kinesthetic sense or deep sensation, refers to the sense of position, movement, and vibration from muscles, tendons, joints, and other motor organs. Proprioception plays a critical role in postural control. A previous study has shown that proprioceptive training could improve sensory activities in the brain of stroke patients with poor functional recovery, aiding in the restoration of balance and motor function (Dechaumont-Palacin et al., 2008). There are various methods for proprioceptive stimulation (PS), which mainly include traditional vibration training, balance training, and rotational training (Heo et al., 2020, Kim et al., 2022, Xu et al., 2024). Whole-body vibration (WBV) is one of the simplest forms of proprioceptive training. It involves stimulating the body with mechanical vibrations, inducing responses in muscles, bones, joints, and the nervous system. Subsequently, this activates muscle stretch reflexes and promotes muscle contraction to enhance muscle strength (Delafontaine et al., 2019). WBV has a positive impact on balance and gait functions in patients with stroke (Sade et al., 2020a, Sade et al., 2020b, Thompson et al., 2020), and has been widely used in the rehabilitation of post-stroke balance disorders in recent years (Yin et al., 2022). However, whether WBV has a positive impact on cortical activation in patients with post-stroke balance disorder remains unknown.

Additionally, it is noteworthy that patients with post-stroke balance disorders often experience vestibular dysfunction, leading to decreased or lost vestibular sensation (Tong et al., 2020). As the vestibular system is responsible for sensing motion and changes in acceleration, its impairment can similarly affect body balance, resulting in symptoms such as motor incoordination (Chien et al., 2007). Therefore, restoring vestibular function is also crucial in addressing balance disorders in patients with stroke. Galvanic vestibular stimulation (GVS) has been shown to induce beneficial neuroplasticity in central pathways of individuals with vestibular loss and has been used in studying self-motion perception and balance control (Lopez & Cullen, 2024). GVS involves applying mild electrical currents to the mastoid processes behind the ears to activate the vestibular nerves, thereby influencing brain activity and function. In patients with post-stroke balance disorders, GVS can activate residual vestibular function, promoting balance recovery (Xie et al., 2024). For instance, the noisy GVS significantly improved postural control during and after stimulation (Inukai et al., 2020). However, few studies have focused on cortical activation in patients with stroke during GVS.

Previous studies used several methods to assess the recovery of balance function in patients with stroke, including the Fugl-Meyer assessment, the Berg Balance Scale (BBS), the timed up and go test, and various balance testing instruments (Sade et al., 2020a, Sade et al., 2020b, Wei and Cai, 2022). However, these evaluation methods lack objectivity. With the development of neuroimaging technologies, objective cortical activation has demonstrated advantages in assessing balance function recovery. Functional magnetic resonance imaging (fMRI) is widely used to assess the impact of GVS on cortical activity (Becker-Bense et al., 2020). Nevertheless, fMRI has certain limitations, which include only measuring brain connectivity in a resting state and being unable to capture real-time cortical activation during task performance. The recently developed functional near-infrared spectroscopy (fNIRS) technology could effectively address these limitations. By attaching optical probes to the scalp, fNIRS emits and receives near-infrared light to measure the concentration changes of oxyhemoglobin (HbO) and deoxyhemoglobin (HbR) in the cerebral cortex. These measurements reflect hemodynamic changes in the tissues and infer potential neural activity in the brain. fNIRS offers good temporal resolution, real-time detection capabilities, resistance to motion artifacts, and is simple and portable. It allows convenient and objective recording of brain activity during movement or stimulation processes, making it suitable for studying the effects of various brain stimulation techniques on brain function (Balardin et al., 2017, Struckmann et al., 2022). A previous study demonstrated that fNIRS is sufficiently sensitive to evaluate GVS-induced cortical activation (Hernandez-Roman et al., 2023, Hernández-Román et al., 2023).

Neuroplasticity and brain functional reorganization form the basis of post-stroke rehabilitation therapy. External stimulation and functional training can promote neurogenesis, increase axonal and dendritic branching, and enhance cortical activation (Joy and Carmichael, 2021, Siponkoski et al., 2020). Although existing studies have indicated that GVS and PS could improve balance function to varying degrees, the specific central mechanisms by which GVS and PS improve balance function remain unclear. Moreover, to date, there have been no studies utilizing fNIRS to explore the effects of combined GVS and PS on cortical activity. Based on the aforementioned background, this study focuses on using fNIRS to explore the differences and characteristics of cortical activation in healthy individuals and patients with post-stroke balance disorder during GVS, PS, and combined PS and GVS tasks. We hypothesize that all three stimuli will induce cortical activation in healthy individuals, with combined stimulation producing a greater degree of activation, and that this effect will also be observed in patients with post-stroke balance disorders. The study aims to uncover the underlying neurophysiological mechanisms and provide scientific evidence for developing more targeted and effective balance rehabilitation strategies in patients with post-stroke balance disorders.

## 2 Materials and methods

### 2.1 Participants

In this study, G*Power 3.1.9.7 (Kiel University, Kiel, Germany) was used for sample size calculation. Based on the results of our preliminary pilot study, the mean difference and standard deviation (SD) in HbO concentration in the right motor cortex (MC) during combined PS and GVS task and GVS task in healthy individuals were 0.036 and 0.053, respectively. The calculated effect size was 0.67. With an α-level set at 0.05 and a power of 0.80, the final sample size was calculated to be 16 for health participants. We hypothesized that patients with post-stroke balance disorders also demonstrated this level of difference in these task performances, meaning that each group required at least 16 participants. All participants were recruited from the Department of Rehabilitation Medicine, the Second Affiliated Hospital of Guangxi Medical University, between December 2022 and November 2024. A total of 16 patients with stroke (patient group) and 20 healthy controls (healthy group) were ultimately enrolled in the study. The study was reviewed and approved by the Medical Ethics Committee of the Second Affiliated Hospital of Guangxi Medical University (approval number: 2024-KY (0868)). All participants were aware of the purpose and procedures of the study and signed an informed consent form before recruitment.

The inclusion criteria were as follows: (1) Participants in the patient group met the diagnostic criteria of the Chinese Clinical Diagnostic and Treatment Guidelines for Cerebrovascular Disease (second edition) and were diagnosed with ischemic stroke by cranial CT or MRI, with the lesion located in the unilateral hemisphere of the brain (Liu et al., 2023); The duration of the disease ranges from 2 weeks to 6 months; BBS scores between 21 and 40; (2) Aged 40–70 years (Mori et al., 2022); (3) Right-handed; (4) Montreal Cognitive Assessment (MoCA) scores ≥26 (Cui et al., 2023); (5) Willing to comply with the experimental procedures and sign the informed consent form.

Exclusion criteria were as follows: (1) Cranial defects, head infections, or skin lesions that prevent fNIRS imaging; (2) Severe cardiac problems, neurodegenerative diseases, epilepsy, peripheral vascular disease, vestibular disease, severe bone and joint disease, severe osteoporosis, etc; (3) Presence of metal implants; (4) Pregnancy; (5) Inability to tolerate stimulation.

### 2.2 Clinical measurements

Demographic data, including sex, age, height, weight, and Body Mass Index (BMI), were collected for all participants prior to the intervention. Additional clinical characteristics, including disease duration and infarcted hemisphere, were recorded for patients in the study group. Two experienced therapists independently administered both the MoCA and BBS to all participants following standardized protocols. The MoCA is a widely validated neuropsychological screening instrument designed to assess multiple cognitive domains, including sustained attention, working memory, language processing, visuospatial abilities, and executive functioning. It has established clinical utility for detecting mild cognitive impairment in diverse patient populations (Nasreddine et al., 2005). The BBS is a commonly used clinical tool for assessing balance ability, widely employed in elderly individuals and patients with neurological disorders such as stroke and Parkinson’s disease. The BBS consists of 14 items, with each item scored on a range from 0 to 4, based primarily on the completion of the task. The total score ranges from 0 to 56. The scale demonstrates good reliability and validity (Pickenbrock et al., 2016).

### 2.3 GVS

GVS was delivered using a transcranial direct current stimulation (tDCS) device (The Brain Driver tDCS v2.1, TheBrainDriver, LLC, IL, USA) configured for peripheral stimulation. GVS applies microcurrents through electrodes placed on the mastoid behind the ear to simulate the nerve impulses generated by head movements or other stimuli in natural situations. This directly or indirectly activates vestibular receptors and nerve fibers to produce effects similar to natural vestibular stimulation, thus activating the vestibular function that is residual after injury due to stroke and thus facilitating the restoration of the equilibrium of patients (Utz et al., 2010). When a bipolar GVS is applied in the quiet state of a normal human body, the net effect of the stimulus produced is a shift in body posture to the anodic side. Moreover, the cathodic stimulus specifically activates vestibular afferent fibers, especially irregular, spontaneously firing nerve fibers, while the anodic stimulus inhibits vestibular signaling (Stolbkov et al., 2014). When performing GVS, the participants stood on the vibrator platform and wore blindfolds. The researcher placed patches on both sides of their postauricular mastoid, with the anode placed on the postauricular mastoid on the side of the lesion, and the cathode placed on the postauricular mastoid on the contralateral side to the lesion, at a current intensity of 2.0 mA and duration of 30 s (Hernández-Román et al., 2023, Hernandez-Roman et al., 2023, Tohyama et al., 2021).

### 2.4 PS

Proprioception includes positional, kinesthetic, and vibratory senses. WBV is a PS method that is simple and easy to implement. The vibrator passes vibration waves and acts on the body to produce adaptive responses in the nervous system, musculoskeletal system, and cardiovascular system, thereby improving body functions. Stimulation of the human body through mechanical vibration activates the muscle’s tensor reflex, which prompts muscle contraction and strengthens the muscles (Sitjà-Rabert et al., 2015). The study procedure was refined and adjusted based on the literature and through insights from previous experiments. Briefly, the participants stood on the platform of the vibrometer (Xiaomi Technology Co., Ltd., Beijing, China), wore blindfolds, stroked the guardrails on both sides with both hands, and relaxed their bodies with the vibration, with the parameter selection on the 1st gear (motor rotation speed of 800 revolutions, amplitude of 8 mm) and duration of 30 s (Choi et al., 2017).

### 2.5 Combining PS and GVS

Participants stood on the vibration platform wearing blindfolds, maintaining light bilateral hand contact with the side rails. Disposable surface electrodes were positioned over both mastoid processes. GVS and PS were administered concurrently.

### 2.6 Experiment procedures

**Fig. 1:**
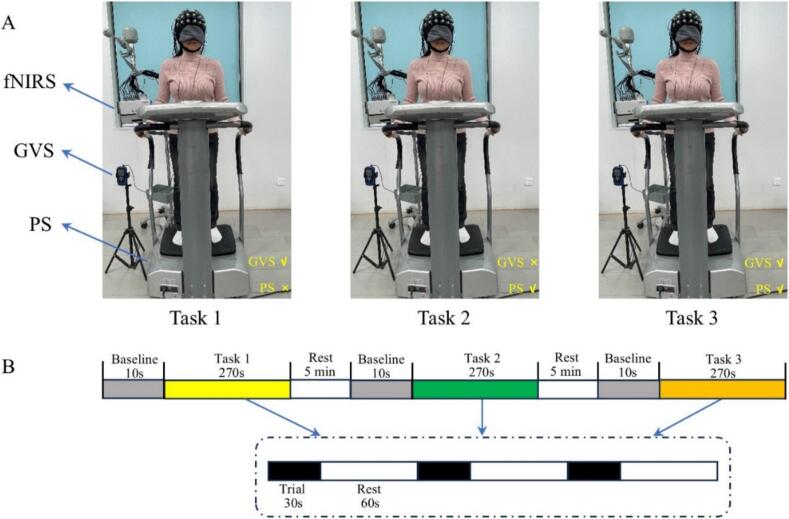
(A) Diagram of three different stimulation tasks. (B) fNIRS measurement procedure. Task 1: receiving only GVS; Task 2: receiving only PS; Task 3: receiving both GVS and PS. fNIRS: functional Near-Infrared Spectroscopy; GVS: Galvanic Vestibular Stimulation; PS: Proprioceptive Stimulation.

The experiment was performed in a treatment room with only the researcher and the participant present. The participants were given a fNIRS spectral data acquisition hat and blindfolds before the start of the experiment, which was designed to minimize the optic-eye-movement reflex and visual-vestibular input (Sasaki et al., 2024). Participants received three different stimulation tasks: Task 1: receiving only GVS; Task 2: receiving only PS; Task 3: receiving GVS and PS (Fig. 1A). Before the formal experiment, the room environment was optimized to ensure appropriate temperature, humidity, adequate lighting, and absence of external noise (Xiao et al., 2025). The tasks were conducted under the supervision of a therapist with many years of experience in physical therapy, who was highly familiar with both PS and GVS, but was blinded to the group allocation. Participants were informed that they would receive three types of stimulation in a concealed order and were instructed to remain relaxed, avoid head movement, and refrain from speaking during the tasks to minimize distractions. The computerized equipment was used to start and end each task automatically with simple but accurate verbal prompts based on the experimental settings. Then, the participants stood bipedally on the platform of the resonator with their hands gently holding the guardrails on both sides. At the beginning of each task, 10 s of baseline data were collected, after which the participants were subjected to the block design paradigm in the following order: 30 s of stimulation followed by 60 s of rest, a pattern that lasted for 3 cycles. After each stimulation task, participants are required to rest for 5 min to allow the HbO concentration to fully return to baseline levels (Fig. 1B). Throughout the experiment, the fNIRS system monitored the HbO concentration in all channels of the participant in real-time. Due to the different sides of the lesions of stroke participants, the data of stroke participants with right-sided lesions were uniformly mirror-flipped (Mihara et al., 2021, Tamashiro et al., 2019). After flipping, the fNIRS data on the affected side of all stroke participants were located on the left side.

### 2.7 fNIRS measurements and data processing

A multichannel fNIRS system (NirSmartll-6000A, Danyang Huichuang Medical Equipment Co., Ltd., Jiangsu, China) was used to record changes in the HbO concentration. The system consists of a near-infrared light source (light-emitting diodes) and avalanche photodiodes as detectors. In this study, 23 light sources and 15 detectors comprised 49 active observation channels covering the cortical region of interest (ROI). Cortical activity was recorded using dual wavelength (730 and 850 nm) with a sampling rate of 11 Hz. The light sources and detectors were fixed using acquisition head caps, and the distance between them was set at an average distance of 3.0 cm.

**Fig. 2:**
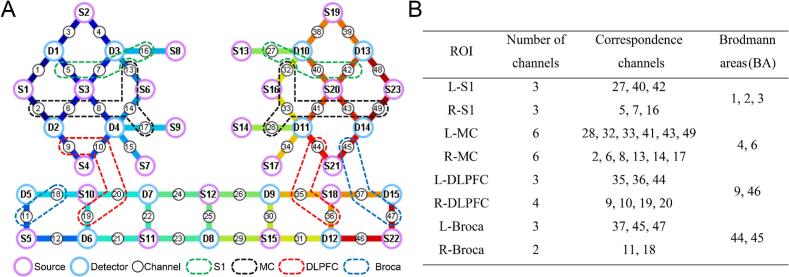
(A) fNIRS light source and detector channel arrangement (purple S is the light source, blue D is the detector). (B) Brodmann area and fNIRS channels included in ROI. (For interpretation of the references to colour in this figure legend, the reader is referred to the web version of this article.)

Based on Brodmann area (Szczepanski & Knight, 2014), the ROIs in this study included the somatosensory cortex (S1), MC, Dorsolateral prefrontal cortex (DLPFC), and Broca’s area (Broca) (Fig. 2A). The left somatosensory cortex (L-S1) consisted of channels 27, 40, and 42. The right somatosensory cortex (R-S1) consisted of channels 5, 7, and 16. The left motor cortex (L-MC) consisted of channels 28, 32, 33, 41, 43, and 49. The right motor cortex (R-MC) consisted of channels 2, 6, 8, 13, 14, and 17. The left dorsolateral prefrontal cortex (L-DLPFC) consisted of channels 35, 36, and 44. The right dorsolateral prefrontal cortex (R-DLPFC) consisted of channels 9, 10, 19, and 20. The left Broca’s area (L-Broca) consisted of channels 37, 45, and 47. The right Broca’s area (R-Broca) consisted of channels 11 and 18 (Fig. 2B).

Data processing was conducted with Nirspark (Nirspark v1.8.1, Danyang Huichuang Medical Equipment Co., Ltd., Jiangsu, China). To remove the raw data containing physiological, instrumental, and other noise, the original data was preprocessed using the following steps. First, removing task-irrelevant artifacts and time period. Second, converting light intensity to optical density and filtering the data in the 0.01 to 0.2 Hz range to remove the task-irrelevant effects of low-frequency drift and high-frequency neurophysiological noise. Third, the optical density was converted to HbO and HbR concentration according to the Beer-Lambert law (Yao et al., 2024). Finally, the HbO concentration of each channel from 5–35 s for each task was superimposed and averaged. Previous studies have shown that HbO signals in near-infrared spectroscopy brain functional imaging exhibit a delayed nature and cannot be detected until 5 s after the stimulus (Xia et al., 2022). The HbO concentration of each ROI during each task reflected the activation of the corresponding ROI.

### 2.8 Statistical analysis

IBM SPSS Statistics version 23.0 (IBM Corp., Armonk, NY, USA) was used to conduct statistical analyses. Continuous variables were presented as mean ± SD ($\overset{¯}{x}$ ± SD), while categorical variables were expressed as frequencies and percentages. The Chi-square test was used to analyze differences in sex between the two groups. Normality of distributions for age, height, weight, MoCA scores, and BBS scores was examined using Shapiro-Wilk tests. An independent *t*-test was conducted for normally distributed data; otherwise, a non-parametric test was applied. For fNIRS, when performing within-group comparisons, data following a normal distribution were analyzed using a paired sample *t*-test, while non-normally distributed data were analyzed using a paired Wilcoxon rank-sum test. When doing between-group comparisons, normally distributed data were analyzed using an independent sample *t*-test, and non-normally distributed data were analyzed using the Mann-Whitney rank-sum test. The false discovery rate method was used for multiple-comparison correction. Missing data was imputed using the multiple imputation method. Significant differences were defined as #/*: *p* < 0.05, ##/**: *p* < 0.01, and ###/***: *p* < 0.001.

## 3 Results

### 3.1 Demographic characteristics

In this study, 16 participants with post-stroke balance disorders and 20 healthy controls were enrolled in the final analysis. As shown in Table 1, there were no significant differences in sex, age, height, weight, BMI, or MoCA scores between the two groups (*p* > 0.05). There was a significant difference in BBS scores (*p <* 0.001), with a mean score of 52.8 in the healthy group and 27.7 in the patient group, respectively.

**Table 1:** General information on participants in the health group and the patient group.

Variable	Health group (n = 20)	Patient group (n = 16)	*p-*value
Sex (M/F)	14/6	13/3	0.925
Age (Years)	51.8 ± 8.1	53.3 ± 8.4	0.590
Height (cm)	165.3 ± 6.8	165.4 ± 7.5	0.959
Weight (kg)	59.7 ± 6.2	58.1 ± 8.7	0.543
BMI (kg/m^2^)	21.8 ± 1.4	21.2 ± 1.7	0.209
MoCA (Scores)	28.6 ± 1.8	27.8 ± 1.9	0.201
BBS (Scores)	52.8 ± 3.5	27.7 ± 6.4	0.000***
Disease Duration (Days)	/	93.4 ± 54.7	/
Infarcted hemisphere (L/R)	/	12/4	/

***p < 0.001.

### 3.2 Activation of ROIs in the healthy group

**Fig. 3:**
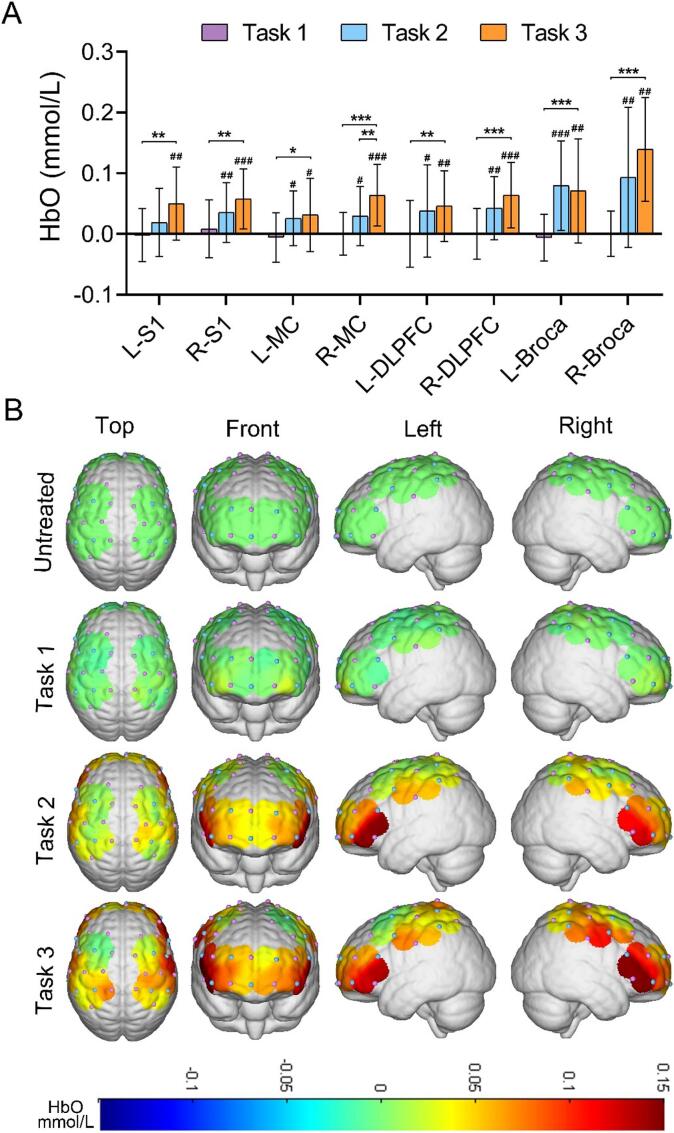
Cortical activation states during different stimulation tasks. (A) Histograms of differences in mean HbO in various brain regions during different stimulation tasks in the healthy group and (B) corresponding cortical activation. #/*p < 0.05, ##/**p < 0.01, ###/***p < 0.001 (The “#” in the figure indicates the stimulation process compared to pre-stimulation, and the “*” indicates the comparison between different stimulation tasks).

First, the mean HbO concentration before and during the stimulation of each ROI in each task was obtained for comparison. As shown in Fig. 3A, the difference between the mean HbO concentration before and during each ROI stimulation for Task 1 was not statistically significant (*p* > 0.05). In Task 2, HbO concentration in R-S1, bilateral MC, DLPFC, and Broca during stimulation was significantly higher than pre-stimulation concentration (*p* < 0.05). Finally, in Task 3, HbO concentration in the bilateral S1, MC, DLPFC, and Broca during stimulation was significantly higher than pre-stimulation concentration (*p* < 0.05). Next, the differences among the three different tasks were compared. As shown in Fig. 3A, Task 3 had significantly higher mean HbO concentration during stimulation than Task 1 in all ROIs (*p* < 0.05). Compared to Task 2, only the R-MC in Task 3 had a significantly higher HbO concentration during stimulation than Task 2 (*p* < 0.05). Fig. 3B showed a three-dimensional brain map of average cortical activation in the brain of the healthy group during the three tasks. The mean HbO concentration across ROIs during different stimulation tasks in the healthy group was shown in Table S1.

### 3.3 Activation of ROIs in the patient group

**Fig. 4:**
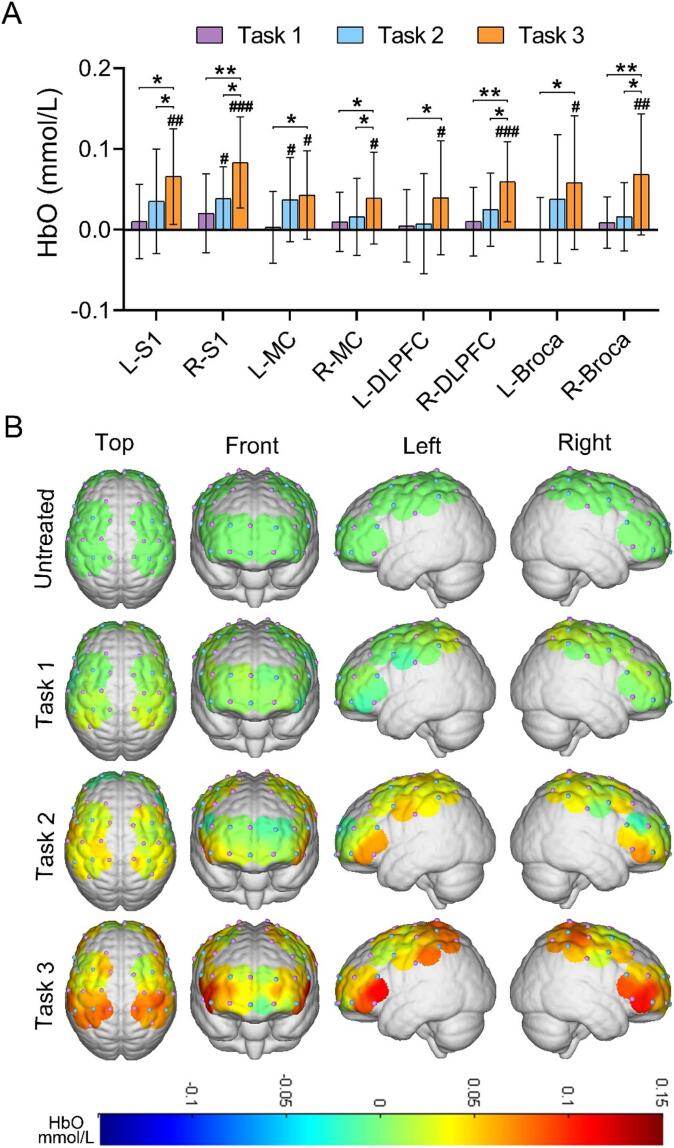
Cortical activation states during different stimulation tasks. (A) Histograms of differences in mean HbO in various brain regions during different stimulation tasks in the patient group and (B) corresponding cortical activation. #/*p < 0.05, ##/**p < 0.01, ###/***p < 0.001 (The “#” in the figure indicates the stimulation process compared to pre-stimulation, and the “*” indicates the comparison between different stimulation tasks).

There was no statistically significant difference among the ROIs in the patient group before and during Task 1 (*p* > 0.05). Regarding Task 2, only R-S1 and L-MC showed significantly higher HbO concentration during stimulation compared to pre-stimulation (*p* < 0.05). In Task 3, HbO concentration during stimulation was significantly higher (*p* < 0.05) than pre-stimulation in all ROIs. We further compared the differences among tasks in the patient group. The mean HbO concentration in all ROIs in Task 3 was significantly higher than in Task 1 (*p* < 0.05). Additionally, the mean HbO concentration in bilateral S1, R-MC, R-DLPFC, and R-Broca was significantly higher in Task 3 than in Task 2 (*p* < 0.05) (Fig. 4A). Fig. 4B showed a three-dimensional brain map depicting the average cortical activation in the patient group during the three tasks. The mean HbO concentration across ROIs during different stimulation tasks in the patient group was shown in Table S2.

### 3.4 Comparison of activation of the ROIs between the healthy and patient groups

**Fig. 5:**
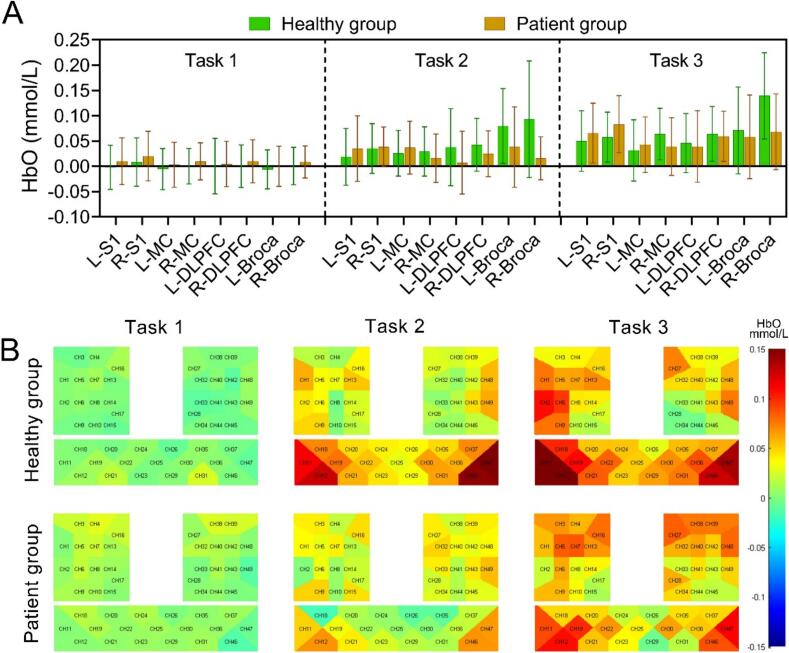
Comparison between the healthy group and patient group. Histograms of mean HbO differences in each ROI for (A) different stimulation tasks and (B) each channel activation process in healthy group and patient group participants.

As shown in Fig. 5A and Table S3, there was no significant difference in HbO concentration of each ROI between the healthy group and the patient group (*p* > 0.05) for Task 1, Task 2, and Task 3.

## 4 Discussion

PS and GVS have been widely used in studies of postural control and balance function (Vecchio et al., 2024, Xie et al., 2024), but the mechanism by which combined PS and GVS induce cortical activation in healthy individuals and people with post-stroke balance disorder remains unknown. Our study explored the activation of each ROI during three different tasks in both populations. Through intra-group and inter-group comparisons of cortical activation, some novel findings were obtained in the present study, which will be discussed in detail below.

In the present study, comparative analyses in each ROI between the healthy group and the patient group before and during Task 1 revealed no statistically significant differences. These observations align with existing neurophysiological evidence showing absent cortical HbO modulation during subthreshold vestibular stimulation (Valdés et al., 2021). This null effect may reflect preserved self-regulatory capacity of the vestibular system in neurotypical individuals, where subthreshold inputs fail to perturb physiological homeostasis (Cheng & Gu, 2018). Notably, the frequency-dependent nature of vestibular stimulation has been well documented. fMRI studies demonstrate that sinusoidal currents at 5 Hz selectively activate the S1, while lower frequencies (1–2 Hz) engage the supramarginal gyrus, thalamic nuclei, and cerebellar-hippocampal networks (Stephan et al., 2005). In contrast, our direct current paradigm likely engages distinct neural mechanisms due to fundamental differences in stimulation physics. Future work should systematically map dose–response relationships between GVS parameters and cortical excitability profiles in intact neurocircuitry. The distributed effects of stroke pathophysiology critically disrupt functional connectivity within integrated neural networks. Post-stroke cognitive impairment has been associated with decoupled activity among S1, DLPFC, and medial prefrontal cortex (mPFC) nodes (Zou et al., 2023). GVS activates vestibular end organs in the inner ear, eliciting action potentials from mechanoreceptors (Utz et al., 2010). These afferent signals are transmitted via the vestibulocochlear nerve to synapse at the vestibular nuclei located in the pontomedullary junction. Following this synaptic relay, a subset of secondary ascending fibers projects to the thalamic nuclei, ultimately reaching the parieto-insular vestibular cortex (PIVC), thereby inducing cortical activation (Kirsch et al., 2016). However, cerebral tissue damage may disrupt the integrity of neural pathways, thereby impairing GVS-mediated signal transmission to the cerebral cortex. Our findings indicate that GVS as a standalone intervention may contribute to suboptimal cortical activation in stroke rehabilitation, necessitating exploration of either more potent modalities or combinatorial treatment approaches.

In the healthy group, the HbO concentration of R-S1, bilateral MC, DLPFC, and Broca during Task 2 was significantly higher than that before treatment. These hemodynamic changes indicate that PS effectively recruits neural networks involved in sensorimotor integration and cognitive processing (Cuppone et al., 2016, Hamer et al., 2025). Mechanistically, musculoskeletal movement generates proprioceptive afferents that ascend via the dorsal column-medial lemniscus pathway to S1, with parallel activation of MC for motor planning (Lopez et al., 2017, Wei et al., 2018). This dual activation pattern confirms the capacity of PS to engage integrated sensorimotor pathways critical for movement regulation. The cognitive dimensions of PS are further evidenced by DLPFC engagement, a hub for executive functions including working memory, attentional modulation, and cognitive flexibility (Baumert et al., 2020, Joyce et al., 2024). Neurostimulation studies corroborate DLPFC's postural control contributions, where targeted neuromodulation of this region enhances balance performance through fronto-cerebellar network facilitation (Lattari et al., 2017). Concurrent Broca activation suggests PS may access motor cognitive encoding systems, given this region's established role in complex movement representation and multisensory integration (Clerget et al., 2011, Clerget et al., 2009).

In the patient group, there was a significantly higher HbO concentration in the R-S1 and L-MC during Task 2 compared to baseline, contrasting with the widespread cortical recruitment observed in the healthy group. The selective activation of R-S1 may reflect partially preserved proprioceptive processing capacity in stroke survivors. The MC mediates real-time gravitational counteraction during static postural control and dynamic reaching movements via corticospinal pathways, which coordinate spatially tuned activation of axial and appendicular musculature to maintain upright equilibrium (Hardesty et al., 2023). In our study, elevated HbO concentration in L-MC may indicate efficient compensatory recruitment of perilesional motor networks. Previous studies showed the localized activation in L-MC may represent an attempt to bypass damaged corticospinal tracts via alternative circuits, but such efforts were constrained by the limited capacity of the brain for functional reorganization in chronic stroke (Dancause & Nudo, 2011). Overall, the results suggested that the impaired brain function of the patient group limited the transmission and integration of proprioceptive information in the brain, and was unable to comprehensively mobilize multiple sensorimotor and cognitively related brain regions as in the healthy group.

In the healthy group, the HbO concentration of each ROI during Task 3 was significantly higher than that before the task. Moreover, in the within-group comparison, the HbO concentration in each ROI during Task 3 was significantly higher than that during Task 1, and significantly higher than that of R-MC during Task 2. The combined stimulation triggered a significant increase in HbO concentration in more ROIs, indicating that this stimulation pattern significantly elevated cortical blood flow. When performing Task 3, information from the vestibular and proprioceptive systems was integrated in the brain. This integration may strengthen the functional connectivity among different ROIs, making the neural pathways of S1, MC, DLPFC, and Broca more active and facilitating the transmission and processing of information. A previous study showed that the vestibular and proprioceptive systems did not operate in isolation but are interrelated and interact with each other in the brain (Akay & Murray, 2021). Exogenous GVS may enhance postural control by correcting aberrant vestibular input signals through its integration into central neural pathways (Stephan et al., 2009). Meanwhile, PS exerts therapeutic effects by activating the intrinsic proprioceptive-visual network of the body, thereby reducing inhibitory interference in central sensory processing (Bösner et al., 2018). This interaction may fundamentally explain why the combined intervention demonstrated superior cortical activation compared to isolated sensory stimulation.

In the patient group, the HbO concentration of each ROI during Task 3 was significantly higher than that before the Task. In the between-task comparison, the HbO concentration during Task 3 was significantly higher than that during Task 1 in each ROI and was significantly higher than that during Task 2 in bilateral S1, R-MC, R-DLPFC, and R-Broca, suggesting that combined stimulation also showed a significant advantage in the patient group. For patients with post-stroke balance disorders, GVS may help to activate some potential vestibular-related neural pathways, which provide a better spatial orientation and balance basis for the effective integration of proprioceptive information. Electrophysiological evidence demonstrated that GVS modulated vestibulospinal reflex pathways by lowering detection thresholds of primary afferent neurons (Keywan et al., 2018, Wuehr et al., 2018). Combined stimulation may simultaneously activate neural pathways related to both systems, allowing their complementary mechanisms to synergistically enhance information exchange and multisensory integration.

There was no significant difference in HbO concentration of each ROI between the two groups in the three different tasks. This phenomenon suggested that the healthy group and the patient group exhibited similar brain neuroregulatory mechanisms when exposed to these different tasks. Although the overall degree of change did not reach the level of significant difference, numerically, the HbO concentration was higher in the healthy group. This may be due to the impaired brain function effect in patients with post-stroke balance disorders, whose nerve conduction efficiency and central integration were limited (Mu et al., 2019), resulting in a lower degree of cortical activation than that of the healthy group under the same stimulation.

This study had some limitations. First, this study limited the age of the participants to 40–70 years old, which focused on the high prevalence group of the disease, but the extrapolation of the findings to other age groups was limited. Further studies should expand the age coverage to improve the generalizability of the results. Second, this study only used the fNIRS technique to assess central performance and lacked multimodal data integration. Future studies will incorporate multidimensional assessment tools such as pressure plate analysis systems and fMRI to better explore central and peripheral mechanisms. Third, this study mainly focused on the short-term central performance of combined stimulation. In the future, more long-term intervention trials should be conducted to further investigate the long-term efficacy and mechanisms of GVS combined with PS treatment.

## 5 Conclusions

This study investigated the cortical activation of GVS, PS, and combined PS with GVS in healthy individuals and patients with post-stroke balance disorders using fNIRS. The results showed that GVS alone did not significantly activate ROIs in both groups. PS alone activated R-S1, bilateral MC, DLPFC, and Broca in the healthy group, while in the patient group, it activated only R-S1 and L-MC. Combining PS with GVS significantly activated all measured ROIs in both groups. There were no considerable differences in the comparison of inter-group activation. These results suggest that the combined PS with GVS rehabilitation pattern may contribute to greater benefits for patients with post-stroke balance disorders. Further research should explore the efficacy and rehabilitation mechanisms of this combined stimulation approach.

## CRediT authorship contribution statement

**Lixia Liu:** Writing – original draft, Visualization, Methodology, Investigation, Formal analysis, Data curation. **Jingchuan He:** Visualization, Investigation, Formal analysis, Data curation. **Feiyu Nong:** Visualization, Investigation, Formal analysis, Data curation. **Yalin Huang:** Formal analysis, Data curation. **Shijun Huang:** Resources, Formal analysis. **Xiangyu Qin:** Validation, Investigation. **Chongwu Xiao:** Writing – review & editing, Writing – original draft, Supervision, Conceptualization. **Yaobin Long:** Writing – review & editing, Supervision, Project administration, Funding acquisition, Conceptualization.

## Ethics approval

The study was reviewed and approved by the Medical Ethics Committee of the Second Affiliated Hospital of Guangxi Medical University, No. 2023-KY (0868). All participants were aware of all the contents of the study and signed an informed consent form.

## Declaration of competing interest

The authors declare that they have no known competing financial interests or personal relationships that could have appeared to influence the work reported in this paper.

## Data Availability

Data will be made available on request.
