# Supplementary material for: Changes in cortical activation during proprioceptive stimulation and galvanic vestibular stimulation in healthy individuals and individuals with post-stroke balance disorders: A functional near-infrared spectroscopy study
Source: Neuroimage Clin. 2025 Jun 8;47:103822. doi: 10.1016/j.nicl.2025.103822 (PMC12178929; doi:10.1016/j.nicl.2025.103822)
Supplement: Supplementary Data 1 [file mmc1.docx]

**Supplementary materials**

**Changes in Cortical Activation During** **Proprioceptive Stimulation and** **Galvanic Vestibular Stimulation in Healthy Individuals and Individuals with Post-Stroke Balance Disorders: A** **Functional Near-Infrared Spectroscopy Study**

**Table S1**

Mean HbO concentration across ROIs during different stimulation tasks in the healthy group.

| **ROIs** | **Tasks** | **HbO（mmol/L）** | ***p-*value** | | |
| --- | --- | --- | --- | --- | --- |
|  |  |  | **Pre-Stim *vs* During Stim** | **Task 3 *vs* Task 1** | **Task 3 *vs* Task 2** |
| L-S1 | Task 1 | -0.002±0.044 | 0.971 | **0.001**** | 0.078 |
|  | Task 2 | 0.019±0.056 | 0.148 |  |  |
|  | Task 3 | 0.050±0.060 | **0.002##** |  |  |
| R-S1 | Task 1 | 0.009±0.048 | 0.971 | **0.002**** | 0.078 |
|  | Task 2 | 0.035±0.049 | **0.009##** |  |  |
|  | Task 3 | 0.058±0.049 | **＜0.001###** |  |  |
| L-MC | Task 1 | -0.005±0.041 | 0.971 | **0.021*** | 0.293 |
|  | Task 2 | 0.026±0.045 | **0.025#** |  |  |
|  | Task 3 | 0.032±0.060 | **0.03#** |  |  |
| R-MC | Task 1 | 0.000±0.035 | 0.971 | **＜0.001***** | **0.002**** |
|  | Task 2 | 0.030±0.049 | **0.022#** |  |  |
|  | Task 3 | 0.064±0.051 | **＜0.001###** |  |  |
| L-DLPFC | Task 1 | 0.001±0.055 | 0.971 | **0.003**** | 0.293 |
|  | Task 2 | 0.038±0.076 | **0.043#** |  |  |
|  | Task 3 | 0.046±0.058 | **0.003##** |  |  |
| R-DLPFC | Task 1 | 0.000±0.042 | 0.971 | **＜0.001***** | 0.082 |
|  | Task 2 | 0.043±0.052 | **0.005##** |  |  |
|  | Task 3 | 0.064±0.054 | **＜0.001###** |  |  |
| L-Broca | Task 1 | -0.006±0.039 | 0.971 | **＜0.001***** | 0.721 |
|  | Task 2 | 0.080±0.074 | **＜0.001###** |  |  |
|  | Task 3 | 0.071±0.086 | **0.002##** |  |  |
| R-Broca | Task 1 | 0.001±0.037 | 0.971 | **＜0.001***** | 0.078 |
|  | Task 2 | 0.093±0.115 | **0.005##** |  |  |
|  | Task 3 | 0.139±0.085 | **＜0.001##** |  |  |

Note: “#” in the table indicates pre- stimulation and during- stimulation comparisons, and “*” indicates comparisons between different stimulation tasks #/**p*<0.05, ##/***p*<0.01, ###/****p*<0.001.

**Table S2**

Mean HbO concentration across ROIs during different stimulation tasks in the patient group.

| **ROIs** | **Tasks** | **HbO（mmol/L）** | ***p-*value** | | |
| --- | --- | --- | --- | --- | --- |
|  |  |  | **Pre-Stim vs During Stim** | **Task 3 *vs* Task 1** | **Task 3 *vs* Task 2** |
| L-S1 | Task 1 | 0.008±0.041 | 0.627 | **0.013*** | **0.042*** |
|  | Task 2 | 0.031±0.055 | 0.092 |  |  |
|  | Task 3 | 0.061±0.051 | **0.001##** |  |  |
| R-S1 | Task 1 | 0.017±0.044 | 0.627 | **0.007**** | **0.031*** |
|  | Task 2 | 0.034±0.034 | **0.011#** |  |  |
|  | Task 3 | 0.074±0.050 | **<0.001###** |  |  |
| L-MC | Task 1 | 0.002±0.040 | 0.889 | **0.032*** | 0.627 |
|  | Task 2 | 0.030±0.046 | **0.049#** |  |  |
|  | Task 3 | 0.037±0.043 | **0.011#** |  |  |
| R-MC | Task 1 | 0.009±0.032 | 0.627 | **0.032*** | **0.043*** |
|  | Task 2 | 0.013±0.040 | 0.225 |  |  |
|  | Task 3 | 0.034±0.046 | **0.017#** |  |  |
| L-DLPFC | Task 1 | 0.003±0.040 | 0.889 | **0.043*** | 0.083 |
|  | Task 2 | 0.008±0.053 | 0.636 |  |  |
|  | Task 3 | 0.033±0.057 | **0.041#** |  |  |
| R-DLPFC | Task 1 | 0.008±0.038 | 0.627 | **0.009**** | **0.031*** |
|  | Task 2 | 0.022±0.040 | 0.093 |  |  |
|  | Task 3 | 0.051±0.042 | **<0.001###** |  |  |
| L-Broca | Task 1 | 0.000±0.035 | 0.989 | **0.019*** | 0.425 |
|  | Task 2 | 0.029±0.069 | 0.119 |  |  |
|  | Task 3 | 0.045±0.068 | **0.017#** |  |  |
| R-Broca | Task 1 | 0.008±0.028 | 0.627 | **0.009**** | **0.033*** |
|  | Task 2 | 0.008±0.039 | 0.202 |  |  |
|  | Task 3 | 0.053±0.062 | **0.005##** |  |  |

Note: “#” in the table indicates pre- stimulation and during- stimulation comparisons, and “*” indicates comparisons between different stimulation tasks. #/**p*<0.05, ##/***p*<0.01, ###/****p*<0.001.

**Table S3**

Mean HbO concentration in healthy group and patient group during different stimulation tasks: An ROI-based comparison.

| **ROIs** | **Tasks** | **Healthy group** | **Patient group** | ***p-*value** |
| --- | --- | --- | --- | --- |
| L-S1 | Task 1 | -0.002±0.044 | 0.008±0.041 | 0.731 |
|  | Task 2 | 0.019±0.056 | 0.031±0.055 | 0.296 |
|  | Task 3 | 0.050±0.060 | 0.061±0.051 | 0.792 |
| R-S1 | Task 1 | 0.009±0.048 | 0.017±0.044 | 0.731 |
|  | Task 2 | 0.035±0.049 | 0.034±0.034 | 0.084 |
|  | Task 3 | 0.058±0.049 | 0.074±0.050 | 0.472 |
| L-MC | Task 1 | -0.005±0.041 | 0.002±0.040 | 0.731 |
|  | Task 2 | 0.026±0.045 | 0.030±0.046 | 0.760 |
|  | Task 3 | 0.032±0.060 | 0.037±0.043 | 0.792 |
| R-MC | Task 1 | 0.000±0.035 | 0.009±0.032 | 0.731 |
|  | Task 2 | 0.030±0.049 | 0.013±0.040 | 0.300 |
|  | Task 3 | 0.064±0.051 | 0.034±0.046 | 0.472 |
| L-DLPFC | Task 1 | 0.001±0.055 | 0.003±0.040 | 0.804 |
|  | Task 2 | 0.038±0.076 | 0.008±0.053 | 0.296 |
|  | Task 3 | 0.046±0.058 | 0.033±0.057 | 0.792 |
| R-DLPFC | Task 1 | 0.000±0.042 | 0.008±0.038 | 0.731 |
|  | Task 2 | 0.043±0.052 | 0.022±0.040 | 0.131 |
|  | Task 3 | 0.064±0.054 | 0.051±0.042 | 0.792 |
| L-Broca | Task 1 | -0.006±0.039 | 0.000±0.035 | 0.740 |
|  | Task 2 | 0.080±0.074 | 0.029±0.069 | 0.558 |
|  | Task 3 | 0.071±0.086 | 0.045±0.068 | 0.792 |
| R-Broca | Task 1 | 0.001±0.037 | 0.008±0.028 | 0.731 |
|  | Task 2 | 0.093±0.115 | 0.008±0.039 | 0.084 |
|  | Task 3 | 0.139±0.085 | 0.053±0.062 | 0.105 |
